# Soil microbiome dysbiosis and rhizosphere metabolic dysfunction drive continuous cropping obstacles of *Codonopsis tangshen*

**DOI:** 10.3389/fmicb.2025.1628234

**Published:** 2025-07-09

**Authors:** Dabing Xu, Chenglin Peng, Guohan Si, Xiangyu Xu, Shujun Zhao, Chuan You, Wuxian Zhou

**Affiliations:** ^1^Institute of Plant Protection and Soil Fertilizers, Hubei Academy of Agricultural Sciences/National Observation and Experiment Station for Soil Quality, Hongshan, China; ^2^Jiangsu Provincial Key Lab for Solid Organic Waste Utilization, Key Lab of Organic-Based Fertilizers of China, Jiangsu Collaborative Innovation Center for Solid Organic Wastes, Educational Ministry Engineering Center of Resource-Saving Fertilizers, Nanjing Agricultural University, Nanjing, China; ^3^Institute of Chinese Herbal Medicines, Hubei Academy of Agricultural Sciences/Key Laboratory of Biology and Cultivation of Herb Medicine, Ministry of Agriculture and Rural Affairs, Enshi, China

**Keywords:** *Codonopsis tangshen*, continuous cropping obstacles, soil microbiome, soil metabolomics, carbon source utilization

## Abstract

Successive monocropping of *Codonopsis tangshen* causes continuous cropping obstacles, impairing growth, yield, and quality. To investigate the soil environmental and microbial changes caused by these obstacles, we collected both continuous cropping (C-crop) and non-continuous cropping (NC-crop) soil for analysis. We employed high-throughput sequencing, Biolog-ECO microplate, and metabolomics technology to evaluate microbial diversity, community structure, and carbon source utilization efficiency. Compared with NC-crop, C-crop decreased the yield and polysaccharide content of *C. tangshen* by 40.47 and 29.4%, respectively. Continuous cropping significantly altered soil physicochemical properties and metabolomes, driving distinct shifts in microbial community structure and impairing carbon utilization efficiency. Microbial carbon use efficiency was positively correlated with key soil bacteria and fungi. However, their abundance decreased significantly under continuous cropping, ultimately disrupting soil carbon cycling. Moreover, key bacterial (e.g., *Flavobacterium*, *Lysobacter*, *Pseudomonas*, *Burkholderia*) and fungal genera (e.g., *Ophiosphaerella*, *Dactylonectria*, *Humicola*) showed strong correlations with critical soil physicochemical properties, microbial carbohydrate metabolism, and rhizosphere metabolite profiles. The reduced abundance of these microbes disrupted soil nutrient balance and microbial activity, potentially contributing to *C. tangshen* continuous cropping obstacles. This study contributes to the understanding of the mechanisms underlying continuous cropping obstacles in *C. tangshen* and lays the foundation for developing strategies to alleviate these obstacles.

## 1 Introduction

*Codonopsis tangshen* (*C. tangshen*), a medicinal and edible plant belonging to the Campanulaceae family, has been widely utilized in traditional Chinese medicine (Lin et al., 2013). Phytochemical investigations have revealed its abundant bioactive constituents, including polysaccharides, lobetyolin, and alkaloids, which demonstrate immunomodulatory, hematopoietic, antioxidant, anti-aging, and antitumor pharmacological activities (Ma et al., 2023). *C. tangshen* is primarily cultivated in western Hubei, northeastern Sichuan, and northern Guizhou, with a total national planting area of around 20,000 hectares and an annual market value reaching 12 billion RMB. *C. tangshen* industry offers substantial economic benefits and plays a vital role in promoting economic development of alpine areas and rural revitalization. However, continuous cropping obstacles pose a major challenge to the sustainable development of the *C. tangshen* industry (Zhang M. et al., 2021).

Continuous cropping obstacle refers to the stunted growth, reduced yield, and increased pest/disease susceptibility caused by repeatedly planting the same crops in the same field (Zeeshan Ul Haq et al., 2023). This is mainly caused by three factors: (1) autotoxicity, (2) soil nutrient imbalance, and (3) disrupted microbial communities (Xi et al., 2019). A prime example is *Salvia miltiorrhiza*, whose growth is significantly suppressed under continuous cropping regimes (Liu et al., 2020). Research confirms that continuous cropping severely disrupts soil health, causing microbial imbalance, reduced enzyme activity, and long-term soil quality decline (Liu et al., 2021). As evidenced by *Panax quinquefolius* continuous cropping, this practice induces progressive soil acidification, elevates salinity levels, and depletes essential nutrient reserves (Li et al., 2021). Similarly, *Astragalus mongholicus* replanting drives rhizosphere microbiome reorganization, characterized by 23% increase in fungal dominance and selective enrichment of pathogenic *Fusarium* spp. (Zhou Q. et al., 2024). Notably, continuous cropping of *Panax notoginseng* promotes the accumulation of root-exuded allelochemicals, destabilizing microbial communities, stimulating *Fusarium oxysporum* growth, and inhibiting root development (Wu et al., 2017). However, how the rhizosphere microecology changes after continuous cropping of *Codonopsis tangshen* remains unclear.

The rhizosphere microecosystem imbalance constitutes the core mechanism underlying these obstacles (Gao et al., 2021). Specifically, continuous cropping disrupts beneficial microbial consortia essential for pathogen suppression and plant health maintenance (Zhang et al., 2020). Plant-microbe crosstalk is mediated through root exudate dynamics that reciprocally modulate microbial colonization patterns (Korenblum et al., 2020). Notably, *arbuscular mycorrhizal* fungi inoculation enhances root membrane permeability and modifies exudate profiles in *Astragalus* spp. (Yizhu et al., 2019). For instance, actinomycetes contribute to plant stress tolerance via phytohormone and siderophore biosynthesis (Sadeghi et al., 2017; Singh and Gaur, 2017). Microbial carbon use efficiency (CUE) affects the fate and storage of carbon in terrestrial ecosystems. High-CUE microbial communities promote greater soil organic carbon storage (He et al., 2024). However, the effects of continuous cropping on the *C. tangshen* rhizosphere soil microbial community structure, carbon source utilization efficiency, and metabolomic characteristics remain rarely reported.

Through integrated field investigations and controlled experiments in Banqiao Town, Enshi City, this study combines microbial community profiling with functional metabolic analysis to systematically evaluate the impacts of continuous cropping on *C. tangshen*. By characterizing shifts in rhizospheric microbiota, carbon metabolism, and soil physicochemical properties, we elucidate the key biotic and abiotic factors limiting *C. tangshen* productivity in continuous cropping systems. This work advances mechanistic insights into cropping obstacles and provides a scientific basis for targeted remediation strategies, which was beneficial for restoring soil health and sustaining *C. tangshen* cultivation.

## 2 Materials and methods

### 2.1 Study site description

The field experiment was conducted in Banqiao Town, Enshi City, Hubei Province, China (30°32′16″N, 109°12′45″E). This region is characterized by a subtropical humid monsoon climate, with mean annual temperature and precipitation measuring 16.2°C and 1,600 mm, respectively. The experimental site features yellow-brown soil with a pH of 4.16 and bulk density of 1.2 g cm^−3^. Prior to experiment initiation, two distinct land-use histories were identified: one plot was subjected to 7 years of continuous corn production prior to 3 years of *C. tangshen* cultivation (designated as continuous cropping, C-crop), while the other plot had been under corn cultivation for the preceding decade (designated as non-continuous cropping, NC-crop).

### 2.2 Experimental design

The experimental trial was conducted from November 2021 to September 2023, incorporating two distinct treatments: continuous cropping (C-crop, continuous cultivation of *C. tangshen*) and non-continuous cropping (NC-crop, featuring maize as the preceding crop). A standardized planting density of 25 cm × 7 cm was implemented across all plots. The experimental design employed four randomized replicates per treatment, with individual plot dimensions measuring 10 m^2^. Fertilization protocols and agronomic management practices were uniformly administered in accordance with conventional local agricultural procedures.

### 2.3 Sample collection and preparation

The sampling protocol consisted of establishing 1 m^2^ quadrats with uniform growth vigor within each experimental plot. The fresh roots of *C. tangshen* were harvested and underwent thorough cleansing and desiccation processes to enable subsequent yield quantification and phytochemical characterization of *C. tangshen*. Concurrently, rhizosphere soil fractions were mechanically separated from bulk soil matrices through controlled manual agitation. Adjacent C-crop and NC-crop rhizospheric zones were systematically sampled using a five-point transect approach, collecting 10 intact root systems per sampling node. Soil samples from each cropping system were sieved (2 mm), homogenized, and analyzed for nutrients, metabolome, and microbiome.

### 2.4 Determination of plant indicators and soil properties

Yield estimation was based on the total fresh weight of *C. tangshen* roots harvested from a defined unit area. The fresh weight per plant was obtained by measuring the fresh weight of 15 plants and calculating the average value. Dried roots were crushed for determination of polysaccharide (Yang et al., 2024) and lobetyolin (Zhao et al., 2016). Air-dried soil samples were analyzed for physicochemical properties using standard agrochemical methods (Bohn et al., 2002). Specifically, soil pH and electrical conductivity (EC) were measured using a potentiometer after shaking for 30 min at a soil-to-distilled water ratio of 1:2.5. Soil organic matter (SOM) was determined using the potassium dichromate method. Total phosphorus (TP) was quantified using the sodium hydroxide melting method, total potassium (TK) by flame photometry, and total nitrogen (TN) by the sulfuric acid digestion Kjeldahl method. Available nitrogen (AN) was measured by converting it to ammonia. Available phosphorus (AP) was determined by leaching with sodium bicarbonate solution followed by the molybdenum-antimony colorimetric method. Available potassium (AK) was extracted with ammonium acetate and measured using flame photometry. Exchangeable calcium (ECa) and magnesium (EMg) were determined by ammonium acetate extraction and flame photometry. Available zinc (AZn) in the soil was measured by DTPA extraction and flame atomic absorption spectrophotometry, and available boron (AB) was determined by boiling water extraction and curcumin colorimetry.

### 2.5 DNA extraction, PCR amplification of 16S rRNA genes and ITS

Microbial DNA was extracted from 0.5 g of soil using the DNeasy PowerSoil Kit (QIAGEN, Inc., CA, United States) according to the manufacturer’s instructions. The concentration and quality of the DNA samples were assessed using a NanoDrop 2000 spectrophotometer (Thermo Scientific, Waltham, MA, United States). Bacterial and fungal sequencing libraries were constructed following the MiSeq Reagent Kit Preparation Guide (Illumina, San Diego, CA, United States) as previously described (Caporaso et al., 2012). The paired-end amplicons were sequenced on an Illumina MiSeq platform (Illumina, San Diego, CA, United States) according to standard protocols at Magigene Technology Co., Ltd. (Guangdong, China). The bacterial 16S rRNA gene V4–V5 hypervariable region (Sun et al., 2013) was amplified using the forward primer 515F (5′-GTGCCAGCMGCCGCGGTAA-3′) and the reverse primer 907R (5′-CCGTCAATTCMTTTRAGTTT-3′), each containing a variable 12-bp barcode sequence. The fungal ITS1-1 region (Mbareche et al., 2020) was targeted using the forward primer ITS5-1737F (5′-GGAAGTAAAAGTCGTAACAAGG-3′) and the reverse primer ITS2-2043R (5′-GCTGCGTTCTTCATCGATGC-3′).

The sequencing data were processed by USEARCH (Zhou Y. et al., 2024) and VSEARCH (Rognes et al., 2016). First, the “vsearch --fastq_mergepairs” script was employed to merge paired-end sequences. The “vsearch --fastx_filter” script was used to clip primers. The “vsearch --derep_fulllength” script was applied to identify unique sequence reads. The “usearch -unoise3” script was utilized to generate amplicon sequence variants (ASVs). The “vsearch --usearch_global” script was used to create an ASV table. The “vsearch --sintax” script, in conjunction with the RDP taxonomic database, was adopted for annotating representative sequences. A standardized number of sequences were randomly extracted from each sample, and alpha diversity indices were calculated using the vegan R package. For taxonomic annotation, representative sequences in the gene catalog were searched against NCBI’s non-redundant protein database with an e-value cutoff of 1 × 10^−5^ using DIAMOND, and the lowest common ancestor (LCA) method was applied to estimate the assignment of genes to specific taxonomic groups. The raw sequencing data have been deposited in the NCBI Sequence Read Archive under BioProject accession PRJNA1133669.^1^

### 2.6 Biolog-ECO technology

This study employed the Biolog-ECO microplate, which contains 31 carbon sources, to analyze the metabolic characteristics of microbial communities, specifically their functional diversity. Initially, soil samples were activated at 25°C for 24 h. Subsequently, 3 g of soil was mixed with 27 mL of 0.85 mol/L NaCl solution and shaken at 200 rpm for 30 min. A 3-mL aliquot of the supernatant was then transferred to 27 mL of NaCl solution and mixed thoroughly. Another 3 mL aliquot of the resulting supernatant was added to an additional 27 mL of NaCl solution, yielding a final dilution of 1:1000. Each well of the ECO plate was inoculated with 150 μL of the diluted solution, with four replicates prepared for each treatment. The inoculated microplate was placed in a humid container and incubated at 25°C. During the incubation period, the absorbance at 590 nm was measured every 24 h using a multi-functional enzyme-linked immunosorbent assay (ELISA) plate reader. The ability of microorganisms to utilize different carbon sources was assessed using the average well-color development (AWCD) index, as described by Garland and Mills (1991). The calculation formula for the AWCD is:

$$\text{AWCD}=\sum_{i=1}^{n}(C_{i}-R)/n$$

*C_i_* is the absorbance value of each reaction well at 590 nm, *R* is the absorbance value of the control well, and *n* is the number of wells. (*C_i_* − *R*) less than 0.06 of wells are calculated as zero (Classen et al., 2003).

### 2.7 Statistical analysis

All experiments are conducted in quadruplicate, and the results were expressed as means ± standard deviations. Statistical analyses were conducted using SPSS 24.0 (IBM Corporation, United States), initiating with one-way analysis of variance (ANOVA) to determine significant differences in *C. tangshen* yield and quality parameters across experimental treatments. Subsequently, a random forest model was constructed using the randomForest package in R, employing variable importance function to identify physicochemical properties linked to *C. tangshen* yield and quality traits. Microbial community analyses were conducted in R (v4.0.3) using the vegan package for α- and β-diversity assessments (Wen et al., 2023). Redundancy analysis (RDA) was employed to evaluate treatment-driven dissimilarities in microbial communities and their correlations with environmental factors. Differential abundance analysis was performed using DESeq2 to identify significantly altered microbial taxa (adjusted *p*-value <0.05). Additionally, correlation networks were constructed with the Hmisc package to explore relationships between key microbial taxa and critical soil physicochemical properties, differential carbon substrates, and differential metabolites.

## 3 Results

### 3.1 Effects of continuous cropping on yield, quality of *Codonopsis tangshen*, and the soil physicochemical properties

**Figure 1:**
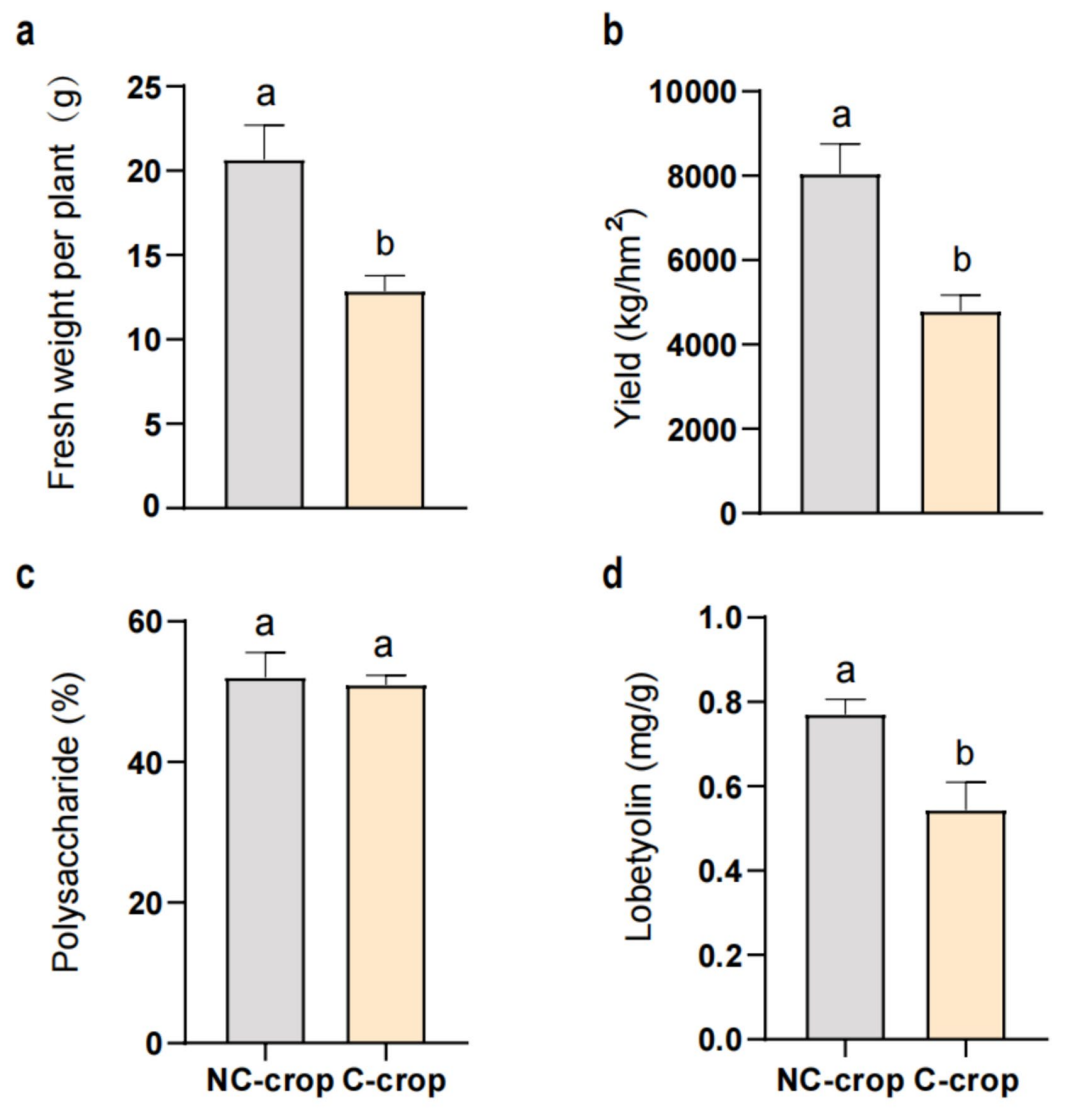
Effect of continuous cropping on the yield and quality of *Codonopsis tangshen*. **(a)** Fresh weight per plant; **(b)** yield; **(c)** polysaccharide content; **(d)** lobetyolin content. C-crop, continuous cultivation of *C. tangshen*; NC-crop, non-continuous cropping of *C. tangshen*.

**Figure 2:**
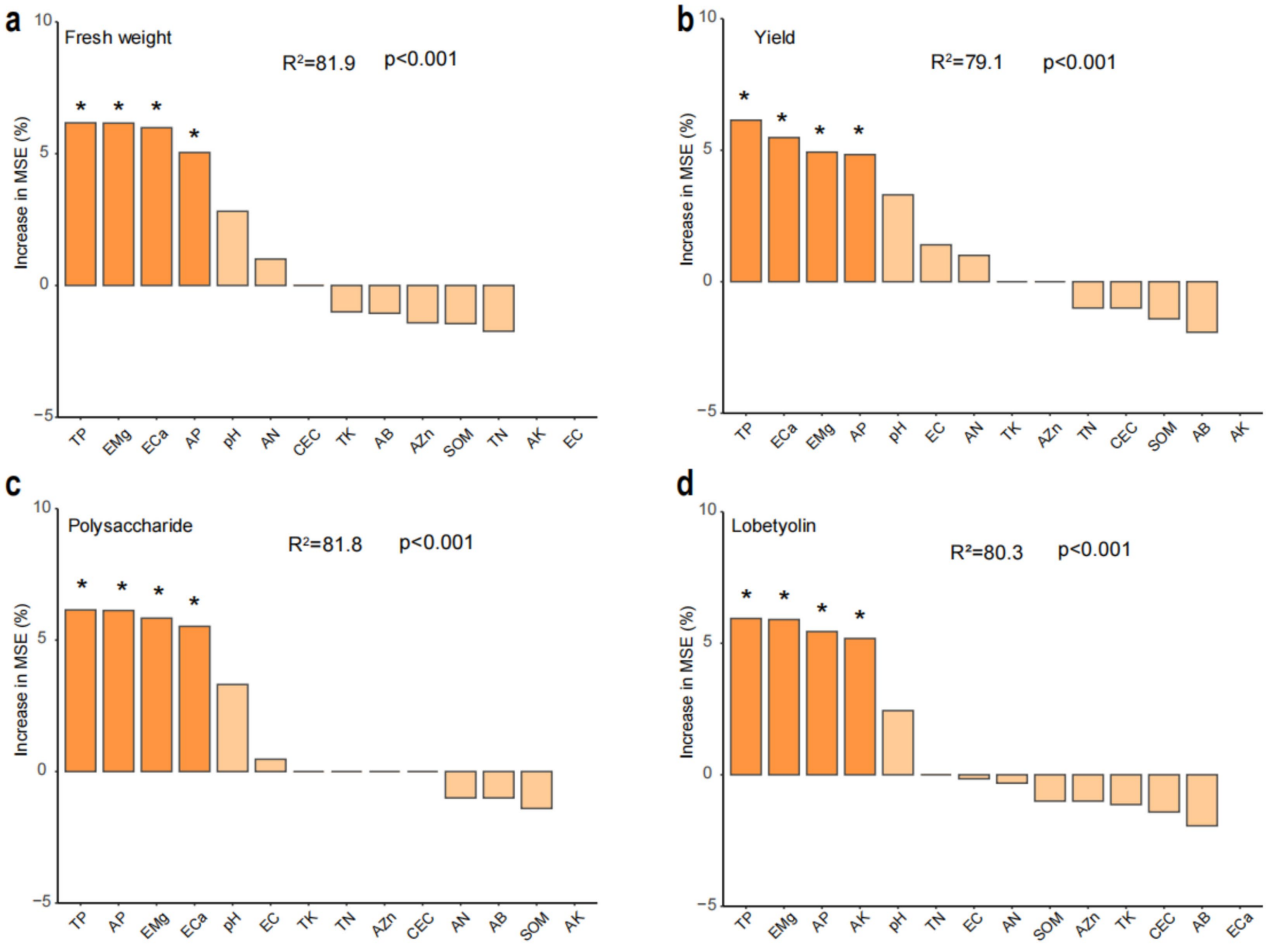
Random forest analysis of potential soil physicochemical factors for changes in yield and quality of *Codonopsis tangshen*. **(a)** Fresh weight per plant; **(b)** yield; **(c)** polysaccharide content; **(d)** lobetyolin. C-crop, continuous cultivation of *C. tangshen*; NC-crop, non-continuous cropping of *C. tangshen*; SOM, soil organic matter; TN, total nitrogen; TP, total phosphorus; TK, total potassium; AN, available nitrogen; AP, available phosphorus; AK, available potassium; ECa, exchangeable calcium; EMg, exchangeable magnesium; AZn, available zinc; AB, available boron; EC, electrical conductivity. The “*” indicates a significant (p < 0.05) explanatory effect on the formation of *C. tangshen* yield and quality.

Significant differential responses in *C. tangshen* growth parameters were observed between C-crop and NC-crop systems (Figure 1). Compared with NC-crop, C-crop cultivation resulted in 37.78 and 40.47% reductions in fresh biomass and yield per plant, respectively. While polysaccharide content remained unaffected by cropping regimes, lobetyolin concentrations exhibited significant depletion under C-crop conditions. Besides, continuous cropping induced soil acidification, evidenced by a 0.89-unit pH decline in C-crop, compared with NC-crop. Concurrently, marked decrease of available potassium (AK, 57.7%), exchangeable calcium (ECa, 88.2%), and magnesium (EMg, 78.2%) was documented in C-crop, compared with NC-crop. Notably, total phosphorus (TP) and available phosphorus (AP) exhibited inverse concentration trends relative to pH dynamics, demonstrating 18.8 and 86.1% increases under C-crop, respectively (Table 1). Subsequently, we employed a random forest model to evaluate the impact of various physicochemical indicators on different key metrics of *C. tangshen*. The study revealed that TP, EMg, ECa, and AP were critical factors influencing the fresh weight, yield, and polysaccharide content of *C. tangshen*. However, ECa did not significantly affect the content of lobetyolins; instead, AK was identified as the primary influencing factor in this context (Figure 2).

**Table 1:** Soil physicochemical properties.

Item	pH	SOM g/kg	TN %	TP %	TK %	AN mg/kg	AP mg/kg	AK mg/kg	Eca mg/kg	Emg mg/kg	Azn mg/kg	AB mg/kg	EC us/cm	CEC cmol/kg
NC-crop	5.37 ± 0.33	49.97 ± 11.11	3.41 ± 3.22	0.95 ± 0.8	19.21 ± 19.34	264.00 ± 50.05	7.00 ± 7.01	268.38 ± 27.49	1591.18 ± 396.22	74.13 ± 26.67	3.43 ± 1.24	0.23 ± 0.05	62.22 ± 17.10	17.71 ± 2.24
C-crop	4.48 ± 0.29	50.94 ± 5.63	3.46 ± 3.66	1.17 ± 1.12	18.77 ± 18.56	266.67 ± 25.28	50.22 ± 14.02	113.59 ± 57.16	187.20 ± 84.12	16.19 ± 3.51	3.52 ± 0.43	0.2 ± 0.04	51.71 ± 7.62	18.29 ± 1.96
Significance	***			***			***	***	***	***				

SOM, soil organic matter; TN, total nitrogen; TP, total phosphorus; TK, total potassium; AN, available nitrogen; AP, available phosphorus; AK, available potassium; ECa, exchangeable calcium; EMg, exchangeable magnesium; AZn, available zinc; AB, available boron; EC, electrical conductivity. Data in the table are mean ± SE, *n* = 4. Using Duncan’s multiple range test of diversity indices separately, ^***^represents *p* < 0.001.

### 3.2 Differences in microbial community structure

**Figure 3:**
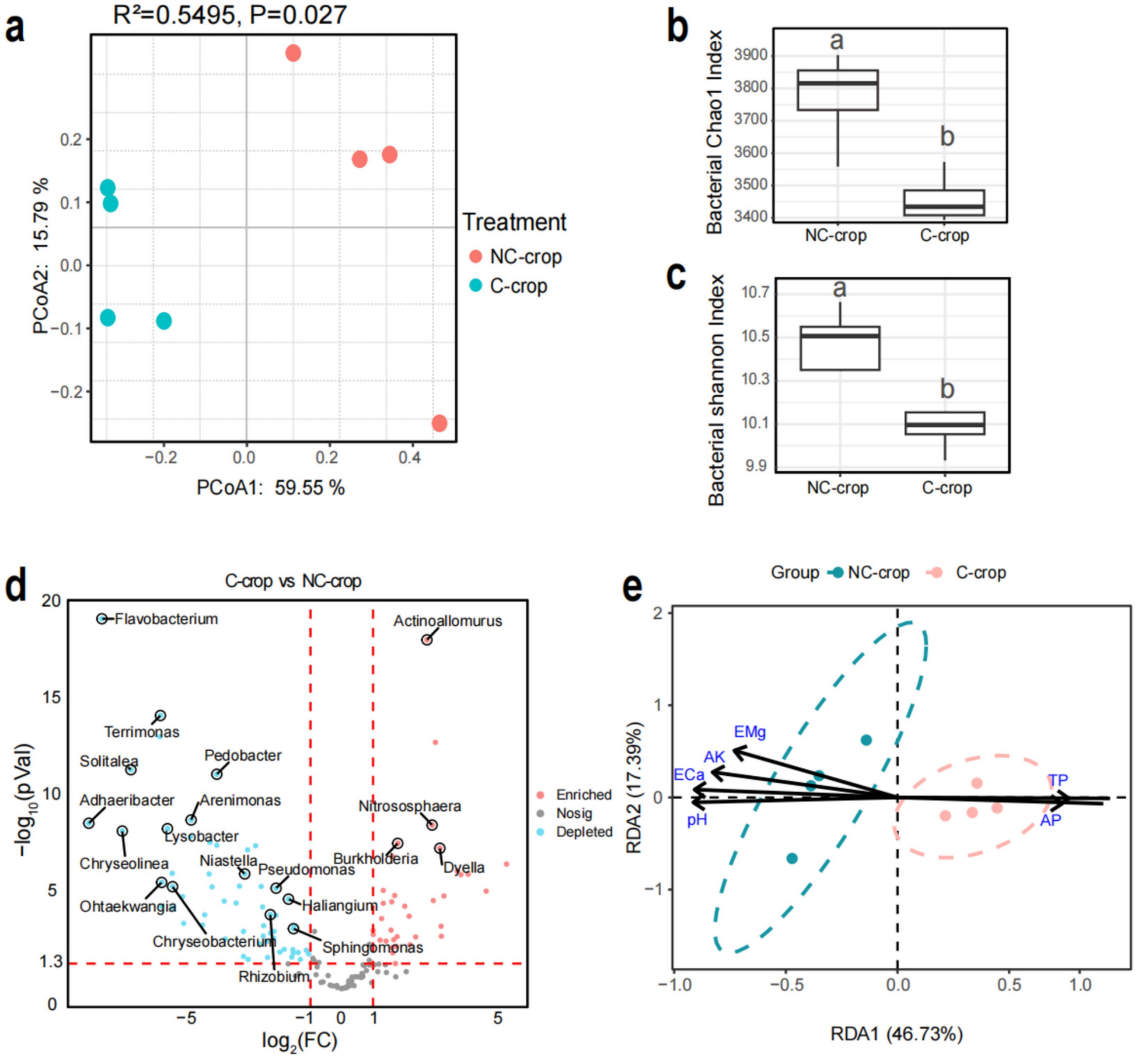
Effects of continuous cropping on bacterial community structure in rhizosphere soil of *Codonopsis tangshen*. **(a)** PCoA analysis of soil bacterial communities. **(b)** Chao1 index of soil bacterial community. **(c)** Shannon index of soil bacterial community. **(d)** Horizontal volcanic map analysis of soil bacterial genera. **(e)** Redundancy analysis (RDA) of bacteria and soil physicochemical properties.

To investigate the effects of long-term continuous monocropping of *C. tangshen* on soil microbial communities, a comparative analysis of bacterial community dynamics was conducted. Principal coordinates analysis (PCoA) revealed distinct separation between the two cultivation treatments (C-crop and NC-crop), demonstrating that *C. tangshen* continuous cropping significantly altered microbial community structure (*R*^2^ = 0.5495, *p* = 0.027). The first two principal coordinates (PCoA1 and PCoA2) accounted for 59.55 and 15.79% of total variance, respectively (Figure 3a). Furthermore, significant reductions in Chao1 richness and Shannon diversity indices were observed in C-crop soils compared to NC-crop controls, indicating that sustained monocropping practices profoundly diminished bacterial diversity (Figures 3b,c). These findings collectively demonstrated that continuous cropping of *C. tangshen* induced substantial structural reorganization and biodiversity loss within soil bacterial communities. Further taxonomic analysis revealed phylum-level alterations, with C-crop soils showing increased relative abundance of Acidobacteria and significant reduction in Bacteroidetes, while other phyla maintained stable proportions (Supplementary Figure S1). Differential abundance analysis at genus level identified 58 genera were significantly downregulated and 35 genera were significantly upregulated in C-crop soil (Figure 3d). The correlation between soil physicochemical factors and soil bacterial communities was analyzed through RDA (Figure 3e). The results indicated that soil physicochemical factors (pH, TP, AP, ECa, EK, and EMg) were important driving factors associated with soil bacterial communities. In addition, soil physicochemical factors like TP and AP significantly affect the composition of microbial communities in continuous cropping soil.

**Figure 4:**
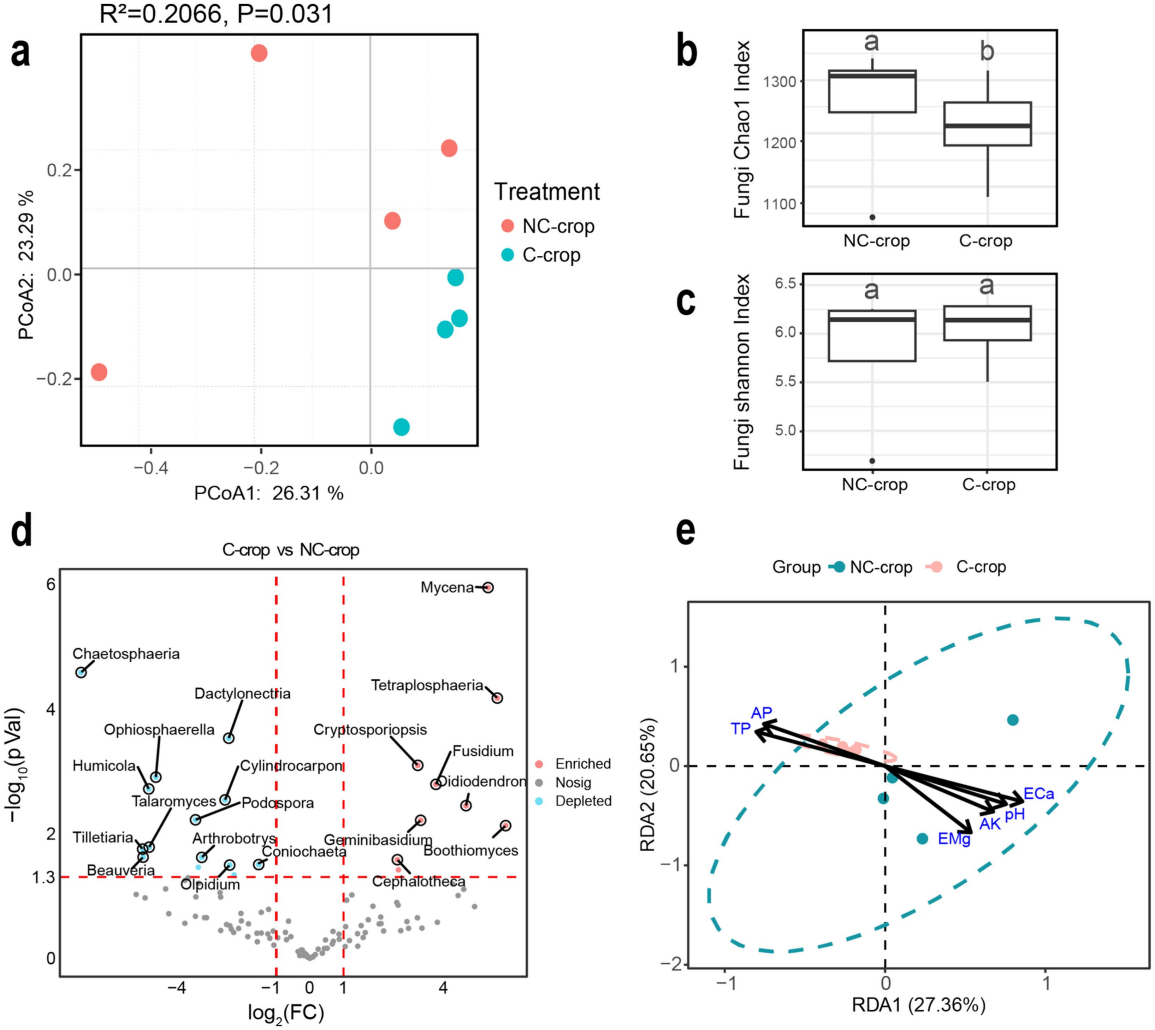
Effects of continuous cropping on fungal community structure in rhizosphere soil of *Codonopsis tangshen*. **(a)** PCoA analysis of soil fungal communities. **(b)** Chao1 index of soil fungal community. **(c)** Shannon index of soil fungal community. **(d)** Horizontal volcanic map analysis of soil fungal genera. **(e)** Redundancy analysis (RDA) of fungi and soil physicochemical properties.

Simultaneously, PCoA ordination revealed significant intergroup differentiation in fungal community composition (*R*^2^ = 0.2066, *p* = 0.031). The principal coordinates (PC1: 26.31% variance contribution; PC2: 23.29% variance contribution) effectively differentiated fungal assemblages across treatments (Figure 4a). Compared to NC-cropt, C-crop significantly reduced soil fungal alpha-diversity as evidenced by decreased Chao1 index (*p* < 0.05), though no significant variation was observed in Shannon diversity index (Figure 4b). Comparative analysis revealed that fungal phyla exhibited more stable relative abundance patterns compared to bacterial phyla across both treatments. Specifically, the C-crop treatment showed increasing trends in Basidiomycota relative to NC-crop controls, while Ascomycota and Mortierellomycota demonstrated significant depletion (Supplementary Figure S2). Furthermore, volcano plot analysis identified 14 fungal genera with significantly downregulated abundance and nine genera with upregulated abundance in C-crop treatment soils (Figure 4c). RDA was employed to elucidate correlations between edaphic factors and fungal community structure. The results demonstrated that, analogous to bacterial communities, TP and AP exhibited significant covariation with fungal community composition in C-crop treatment soils (Figure 4d).

### 3.3 Rhizosphere soil metabolome and microbial carbon source utilization

To mechanistically elucidate microbial community divergence between difference agricultural practices, we performed comparative metabolomic profiling of *C. tangshen* rhizosphere soils under C-crop and NC-crop cultivation regimes. PCA revealed significant metabolic differentiation, with 45.68 and 18.9% variance explained by PC1 and PC2 respectively, cumulatively accounting for 64.58% of total metabolic variation (Supplementary Figure S3a). LEfSe analysis identified treatment-specific biomarker metabolites: NC-cropping soils exhibited preferential accumulation of 26 metabolites, including osmoprotectants (betaine), plant-microbe signaling precursors (hypogeic acid), energy metabolism intermediates (succinic acid semialdehyde), antimicrobial compounds (pelargonic acid), and growth regulators (spermidine). In contrast, C-crop soils uniquely accumulated stress-associated metabolites, notably nitrile derivatives (β-aminopropionitrile), amino aldehyde intermediates (histidinal), histamine analogs (3-methylhistamine), and short-chain fatty acids (valeric acid) (Supplementary Figure S3b). This metabolic dichotomy implies that NC-crop maintains symbiotic microbial networks via growth-promoting pathways, whereas C-crop accumulates stress-response metabolites, potentially reflecting allelopathic inhibition and microbial dysbiosis.

**Figure 5:**
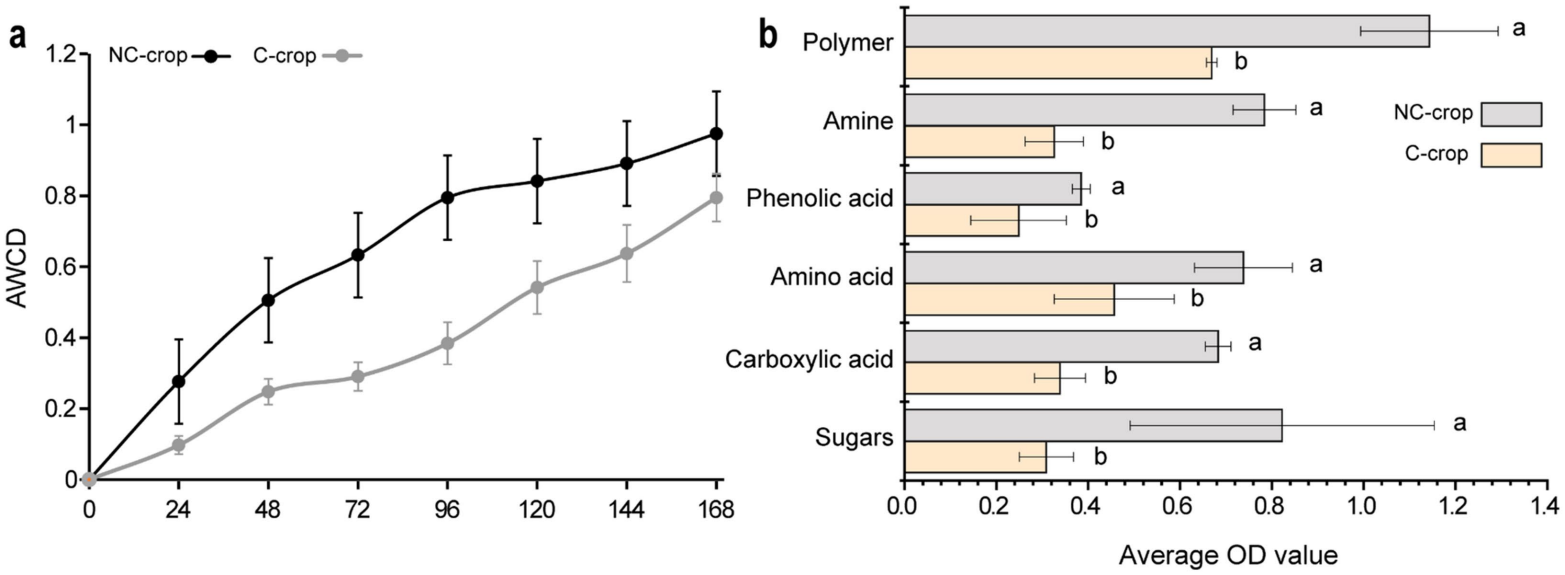
The effect of continuous cropping on the utilization of microbial carbon sources in the rhizosphere of *Codonopsis tangshen*. **(a)** AWCD effects of six carbon sources on the rhizosphere microbial community of *Codonopsis tangshen*. **(b)** Bar chart of differences in absorption of six types of carbon sources by rhizosphere microorganisms of *Codonopsis tangshen*.

The Biolog ECO microplate system serves as a widely adopted methodology for assessing metabolic functional diversity in microbial communities. The average well color development (AWCD) metric provides a quantitative indicator of microbial metabolic activity, with higher AWCD values correlating with elevated microbial functional capacity. As illustrated in Figure 5a, both C-crop and NC-crop treatments exhibited a gradual increase in AWCD values over the cultivation period. Notably, the C-crop treatment demonstrated significantly attenuated AWCD values compared to NC-crop treatment, suggesting substantial suppression of microbial metabolic activity under continuous cropping conditions. Besides, the 31 carbon substrates embedded in Biolog-ECO microplates were categorized into six biochemical classes based on molecular characteristics: polymeric compounds, phenolic acids, amines, amino acids, carboxylic acids, and carbohydrates. Comparative analysis revealed consistently reduced utilization efficiencies across all carbon source categories in C-crop soil relative to NC-crop soil (Figure 5b). The most pronounced differential utilization was observed in carbohydrate substrates, followed sequentially by polymeric compounds and amine-class metabolites, highlighting specific metabolic constraints imposed by continuous cropping practices.

### 3.4 Analysis of the correlation between soil metabolites and microbial communities

**Figure 6:**
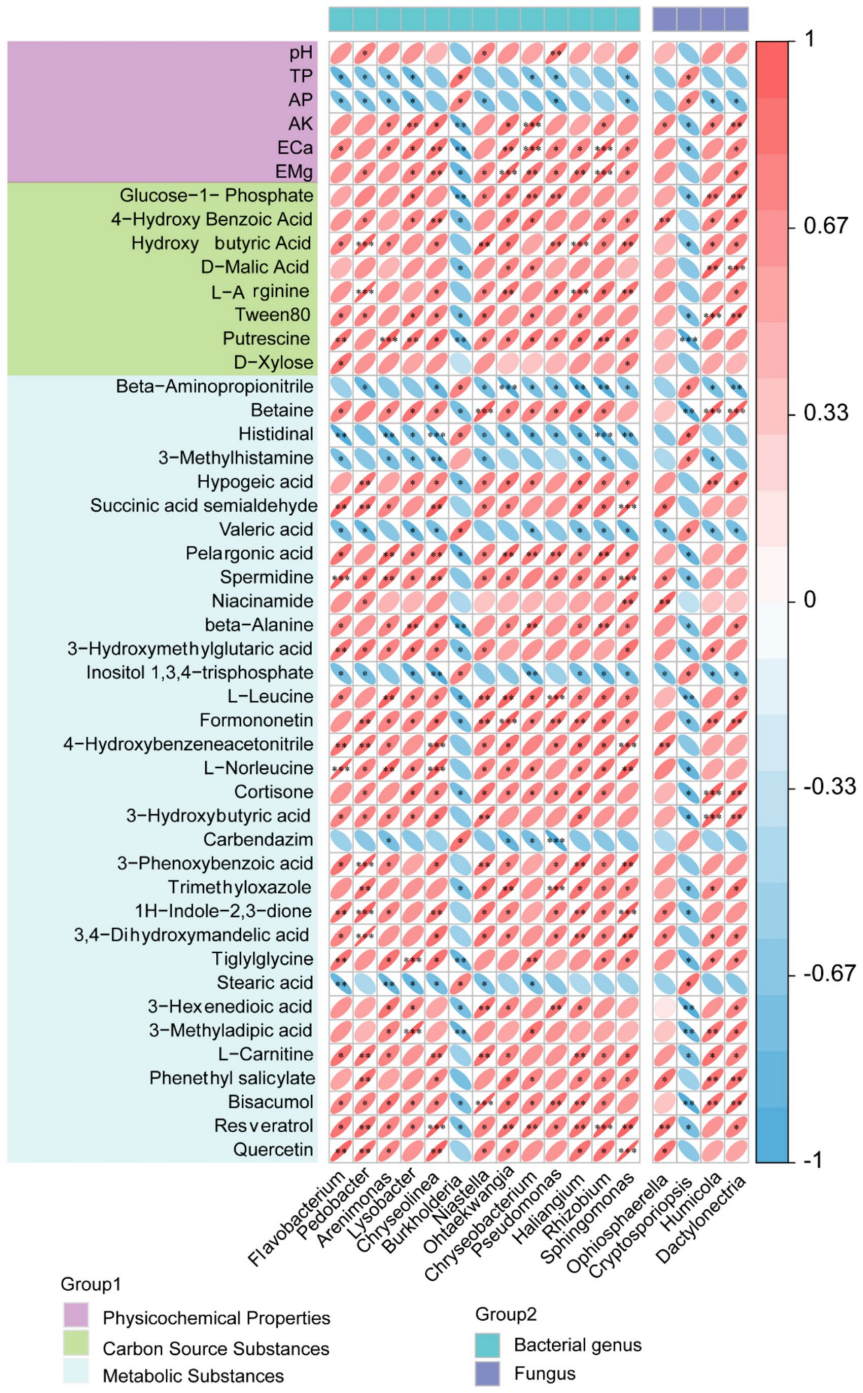
Analysis of the correlation between key physicochemical properties of soil, differential carbon sources, and differential metabolites with key microorganisms.

Multivariate correlation analysis elucidated significant linkages between keystone microbial taxa (13 bacterial and 4 fungal genera), soil physicochemical parameters, carbon source utilization patterns, and metabolite profiles (Figure 6). Soil properties including pH, ECa, EMg, and AK exhibited significant positive correlations with bacterial genera *Flavobacterium*, *Pedobacter*, *Arenimonas*, *Lysobacter*, *Chryseolinea*, *Niastella*, and *Ohtaekwangia*, along with fungal genera *Ophiosphaerella*, *Humicola*, and *Dactylonectria*. Carbon metabolism profiling identified eight substrates with significantly reduced utilization in C-crop soils: glucose-1-phosphate, 4-hydroxybenzoic acid, γ-hydroxybutyric acid, D-malic acid, L-arginine, Tween 80, putrescine, and D-xylose. These carbon sources showed strong positive correlations with the aforementioned microbial taxa, suggesting their functional dependency on specific nutrient acquisition pathways. Conversely, C-crop-enriched metabolites (β-aminopropionitrile, histidinal, 3-methylhistamine, valeric acid, and carbendazim) demonstrated significant negative correlations with the same microbial consortium. This inverse relationship implies metabolite-mediated inhibition of microbial taxa critical for carbon substrate processing, potentially explaining the observed metabolic constraints in continuous cropping systems.

## 4 Discussion

Extensive research demonstrates a progressive decline in soil pH with increasing duration of continuous cropping (C-crop) regimes (Wang B. et al., 2022), coupled with differential nutrient acquisition patterns that fundamentally alter soil nutrient stoichiometry (Li et al., 2022). Our findings reveal severe soil acidification following *C. tangshen* replantation, potentially mediated through two synergistic mechanisms: (1) diminished cation exchange capacity impairing soil buffering against proton accumulation (Zhou et al., 2019), and (2) progressive accumulation of acidic root exudates (e.g., organic acids, phenolic compounds) across successive cultivation cycles (Liu et al., 2013). Concomitant nutrient profile alterations exhibited a paradoxical pattern: marked depletion of cationic macronutrients contrasted with significant phosphorus accumulation. This nutrient imbalance likely stems from crop-specific ion uptake preferences combined with pH-mediated shifts in mineral solubility. The cationic depletion directly compromises soil structural stability through reduced flocculation forces, while phosphorus accumulation may induce ligand competition, further exacerbating micronutrient deficiencies (Yang et al., 2023).

A growing body of evidence establishes soil microbial community composition and diversity as critical determinants of microbially mediated soil functions, with direct implications for soil fertility gradients and agricultural productivity (Muhammad, 2019). Microbial diversity indices serve as ecological barometers, where reduced diversity correlates with diminished ecosystem resilience and compromised sustainability (Xun, 2021). In this study, continuous cropping of *C. tangshen* reduced the *α*-diversity indices of soil bacteria, leading to shifts in bacterial community composition. The bacterial α-diversity indices decreased with the increasing continuous cropping year, which is consistent with the conclusions of previous study (Wang J. et al., 2022). Moreover, the rhizosphere soil bacterial community structure shifted significantly, marked by a pronounced decline in Bacteroidetes abundance and a substantial increase in Acidobacteria abundance. The soil TP and AP have a positive impact on the bacterial community in C-crop soil, while soi pH, ECa, EMg, and AK have a positive impact on the bacterial community in NC-crop soil.

Continuous cropping systems induced significant reductions in fungal community richness as evidenced by diminished Chao1 indices, though fungal diversity (Shannon index) remained statistically unaltered. This pattern aligns with recent findings demonstrating that short-term continuous cropping primarily compromises crop yield through modifications in soil fungal community dynamics (Yu, 2024). Fungal assemblages critically regulate agroecosystem productivity via multifaceted roles in nutrient acquisition, organic matter mineralization, and pathogen-host interactions (Delgado-Baquerizo et al., 2020; Jiang et al., 2021; Hu et al., 2024). Our analysis revealed that prolonged *C. tangshen* continuous cropping substantially changed soil fungal community structure. Redundancy analysis (RDA) of fungal communities demonstrated treatment-specific environmental drivers mirroring fungal community patterns: phosphorus availability (TP, AP) emerged as primary positive correlates in C-crop systems, while cationic nutrients (pH, ECa, EMg, AK) predominantly influenced fungal assemblages under NC-crop conditions. These parallel responses across microbial kingdoms suggest systemic soil restructuring under continuous cultivation, where phosphorus accumulation and cation depletion collectively drive functional microbiome reorganization.

Continuous cropping induces marked declines in soil microbial metabolic activity, fundamentally disrupting microbial-mediated nutrient cycling processes critical for crop nutrient acquisition (Berg and Smalla, 2009). The Average Well Color Development (AWCD), a robust indicator of microbial metabolic potential (Weber et al., 2007), was significantly lower in continuous cropping (C-crop) systems than in non-continuous (NC-crop) controls. This AWCD depression reflects impaired microbial capacity to metabolize diverse carbon substrates, with particularly pronounced reductions in six major carbon source categories: carbohydrates, amino acids, carboxylic acids, polymers, phenolic compounds, and amines. Notably, specific substrate utilization deficiencies emerged in continuous cropping systems, including compromised metabolism of glucose-1-phosphate (glycolytic intermediate), 4-hydroxybenzoic acid (aromatic compound), γ-hydroxybutyric acid (GABA shunt metabolite), D-malic acid (TCA cycle component), L-arginine (nitrogen-rich amino acid), Tween 80 (emulsified lipid source), putrescine (polyamine precursor), and D-xylose (hemicellulose derivative). Carbohydrate metabolism deficits might contribute to impaired energy transduction during decomposition, whereas diminished amino acid utilization may negatively affect microbial nitrogen cycling. Carboxylic acid metabolism limitations, particularly in key TCA cycle intermediates like D-malic acid, suggest systemic energy generation impairments in C-crop microbial consortia (Geisseler, 2014). This metabolic dysregulation creates a self-reinforcing cycle: impaired substrate utilization further restricts microbial functionality, ultimately exacerbating soil health degradation in intensive continuous cropping systems.

Comparative metabolic profiling revealed fundamental biochemical divergence between C-crop and NC-crop cultivation systems. NC-crop soils showed preferential enrichment of organic acids and derivatives, benzenoids, phenylpropanoids, and polyketides, whereas C-crop systems exhibited accumulation of heterocyclic organic compounds. This metabolic shift aligns with the established allelopathic accumulation mechanism, where persistent root exudation of phytochemicals-particularly organic acids-drives rhizosphere microbiome restructuring through pH modification and microbial inhibition, ultimately fostering pathogenic proliferation and crop dysfunction characteristic of continuous cropping obstacles (Wu et al., 2017). Correlation analysis further demonstrated distinct functional associations between microbial communities and soil metabolites across cropping regimes. NC-crop systems maintained robust positive correlations between beneficial bacterial taxa and both carbon source utilization capacity and beneficial metabolite production. Notably, *Flavobacterium*—a genus renowned for high-affinity phosphorus acquisition strategies in phosphorus-limited environments-exhibited stronger metabolic coordination with carbohydrate and organic acid levels. In contrast, C-crop systems showed disrupted microbial-metabolite networks, indicating weakened soil functional connectivity. This microbial-biogeochemical decoupling highlights the metabolic feedback erosion in continuous cropping systems, where declining microbial substrate conversion capacity may worsen soil dysfunction.

Phosphorus-depleted NC-crop soils exhibit selective enrichment of specialized microbial consortia with enhanced nutrient mobilization capacities. *Flavobacterium* thrives under phosphorus limitation by mediating small-molecule carbon metabolism (e.g., monosaccharides and organic acids) and accelerating organic phosphorus mineralization through phosphatase secretion (Ni et al., 2022; Wen et al., 2022; Liu B.-C. et al., 2023). *Lysobacter* demonstrates robust enzymatic potential through extracellular hydrolase production and bioactive secondary metabolite synthesis (Zhao et al., 2021), while *Arenimonas* contributes to soil organic matter turnover via hydrocarbon degradation pathways (Hu et al., 2022). The cellulolytic guild, comprising *Chryseolinea* and *Ohtaekwangia*, drives carbon-nitrogen cycle coupling through coordinated regulation of carbon fixation, methane metabolism, and nitrogen transformation pathways (Zhang S. et al., 2021; Chen et al., 2023). *Niastella* exhibits dual ecological functionality as both a plant-growth promoter and xenobiotic degrader, capable of breaking down complex substrates including cellulose, lignin, and polyaromatic hydrocarbons (Zhao, 2020; Xu et al., 2022). Phosphorus stress adaptations are further mediated by *Chryseobacterium* in acidic soils (Liu B.-C. et al., 2023) and root-mediated recruitment of phosphate-solubilizing *Pseudomonas* (Zhou Q. et al., 2024). Nitrogen cycle modulators include *Haliangium*, which enhances nitrogen fixation and phosphorus mobilization through urease-mediated pathways (Mu et al., 2024), and *Rhizobium*, which directly facilitates atmospheric nitrogen fixation in legume (He et al., 2024) symbioses (Jiang et al., 2021). *Sphingomonas*, belonging to the Proteobacteria, has potential in degrading mulch film materials (Hu et al., 2024). Contrastingly, *Burkholderia* demonstrates negative correlations with soil nutrient parameters while potentially facilitating Basidiomycete nitrogen acquisition through competitive exclusion mechanisms (Liu Y. et al., 2023). Fungal functional specialists such as *Ophiosphaerella* (antimicrobial compound producer) and *Humicola* (cellulose-degrading humification agent) further underscore the microbiome’s role in mediating plant-soil feedbacks, particularly in medicinal plant rhizospheres and composting systems (Wang et al., 2023). This functional specialization underscores the complex interplay between microbial guilds and biogeochemical processes under different agricultural regimes. In this study, the abundance of most microbes mentioned above was significantly lower in C-crop than that in NC-crop, potentially leading to imbalances in soil nutrients and microbial activity, which might contribute to the continuous cropping obstacles observed in *C. tangshen*.

## 5 Conclusion

Continuous cropping of *C. tangshen* induced multifaceted soil degradation, including nutrient imbalance (potassium/calcium/magnesium depletion, phosphorus accumulation), acidification, and microbial community dysregulation. These changes correlated with significant yield reduction and compromised medicinal quality. Continuous cropping conditions restructured the bacterial and fungal communities, characterized by reduced diversity, depleted functional guilds, and pathogen-enriched taxa. These changes collectively impaired carbon/nitrogen cycling and compromised stress resilience. Metabolomic shifts further reflected allelopathic stress and disrupted root-microbe communication. Crucially, soil physicochemical properties (pH, AP, ECa) and microbial carbon use efficiency were strongly associated with keystone genera, including *Flavobacterium*, *Lysobacter*, *Arenimonas*, *Chryseolinea*, *Ohtaekwangia*, *Niastella*, *Chryseobacterium*, *Pseudomonas*, *Haliangium*, *Sphingomonas*, *Burkholderia*, *Ophiosphaerella*, *Dactylonectria*, and *Humicola*, the reduced abundance of these microbes may lead to soil microbiome dysbiosis and rhizosphere metabolic dysfunction, thereby contributing to the continuous cropping obstacles in *C. tangshen*.

## Data Availability

The datasets presented in this study can be found in online repositories. The names of the repository/repositories and accession number(s) can be found in the article/Supplementary material.

## Footnotes

^1^ https://www.ncbi.nlm.nih.gov/
