# Supplementary material for: Soil microbiome dysbiosis and rhizosphere metabolic dysfunction drive continuous cropping obstacles of Codonopsis tangshen
Source: Front Microbiol. 2025 Jul 9;16:1628234. doi: 10.3389/fmicb.2025.1628234 (PMC12283688; doi:10.3389/fmicb.2025.1628234)
Supplement: Supplementary file 1 [file Data_Sheet_1.docx]

**Supplementary materials for**

**Soil microbiome dysbiosis and rhizosphere metabolic dysfunction drive continuous cropping obstacles of *Codonopsis tangshen***

Dabing Xu^1^, Chenglin Peng^1^, Guohan Si^1^, Xiangyu Xu^1^, Shujun Zhao^1^, Wuxian Zhou^2^

**Affiliations**

1 Institute of Plant Protection and Soil Fertilizers, Hubei Academy of Agricultural Sciences / National Observation and Experiment Station for Soil Quality, Hongshan

2 Institute of Chinese Herbal Medicines, Hubei Academy of Agricultural Sciences / Key Laboratory of Biology and Cultivation of Herb Medicine, Ministry of Agriculture and Rural Affairs, Enshi

***Corresponding authors:**

Wuxian Zhou, E-mail: zhouwuxian@hbaas.com.


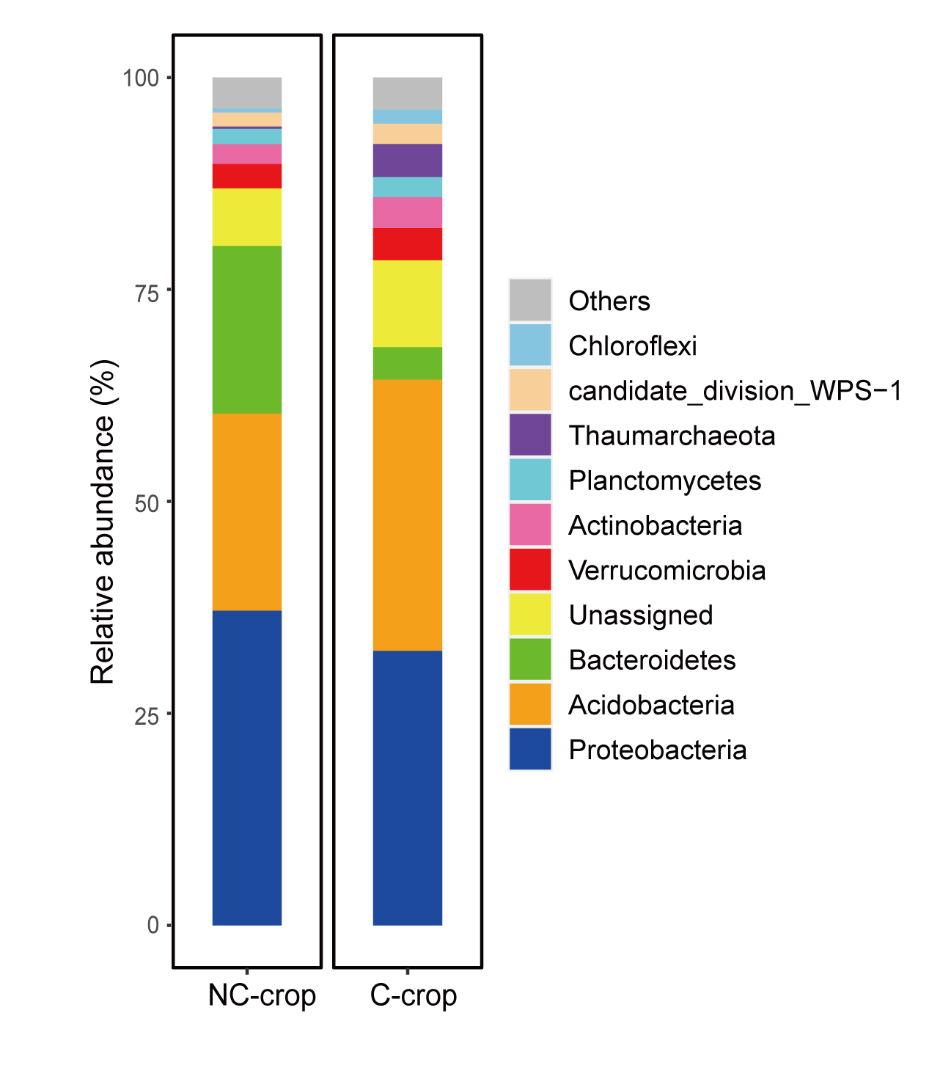


**Fig. S1 The relative abundance of bacterial communities at the phylum level between NC-crop and C-crop treatment.**


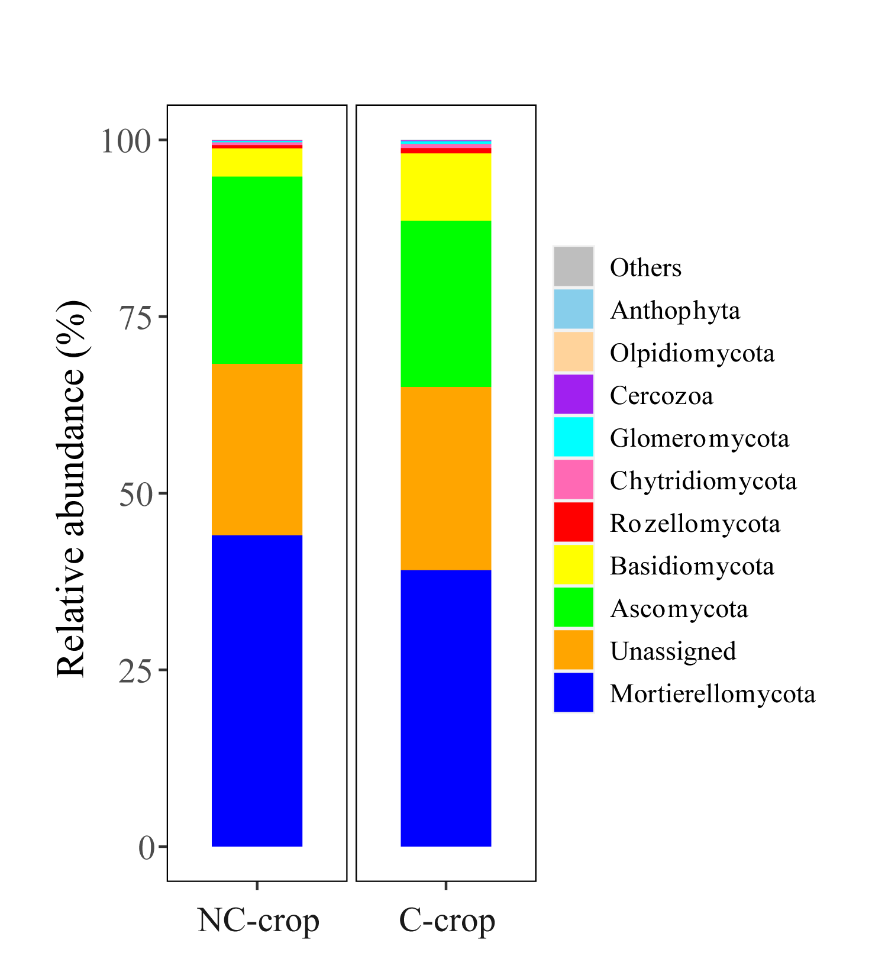


**Fig. S2 The relative abundance of fungal communities at the phylum level between NC-crop and C-crop treatment.**


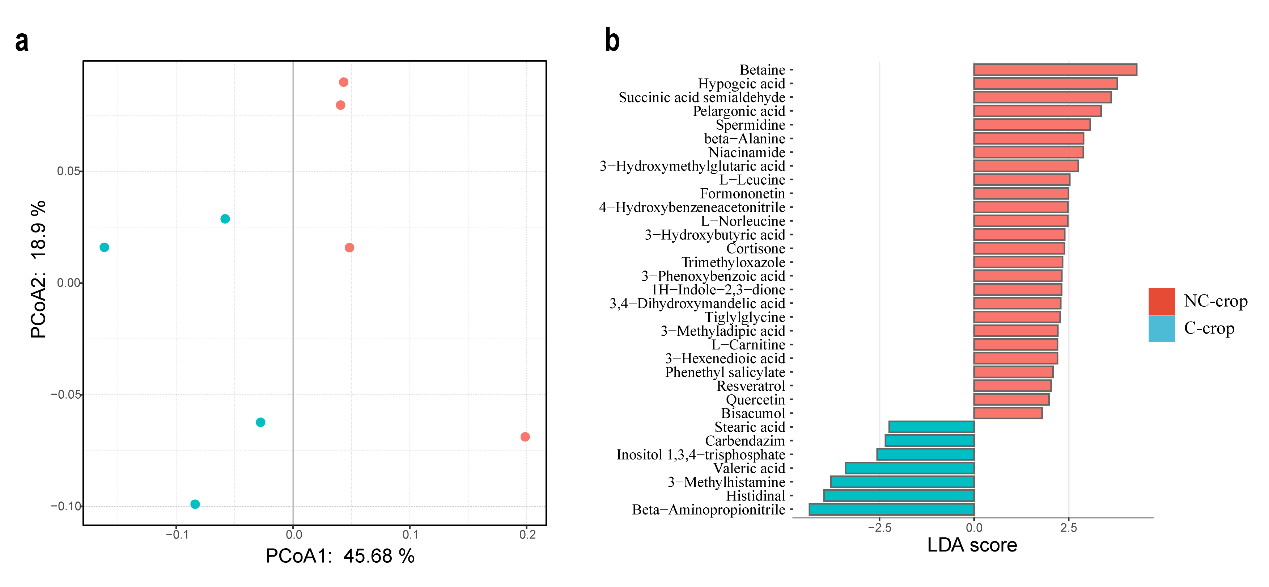


**Fig.S3 The effect of continuous cropping on soil metabolism in the rhizosphere of *Codonopsis tangshen*. (a) principal component analysis of soil metabolism in the rhizosphere of *Codonopsis tangshen*; (b) root zone differential metabolites of *Codonopsis tangshen* between treatments.**
